# “S‐Tau‐Lled” Lysosomes Disrupt Autophagic Processes in Neurodegeneration

**DOI:** 10.1111/tra.70051

**Published:** 2026-09-06

**Authors:** Anaïs Bécot, Mehdi Kabani

**Affiliations:** ^1^ Université Paris‐Saclay INSERM, Neuro‐Bicêtre U1195 Le Kremlin‐Bicêtre France; ^2^ CNRS Protéinopathies du système Nerveux EMR 2000 Le Kremlin‐Bicêtre France

## Abstract

Tauopathies are a class of neurodegenerative diseases characterized by the accumulation of hyperphosphorylated, misfolded and aggregated Tau proteins and by dysfunctions in the autophagy‐lysosome system. Whether the latter are a cause or a consequence of the former is unclear. The answer may come from a recent study by Mirfakhar et al. Using human iPSC‐derived neurons harboring the *MAPT* p.R406W mutation in Tau, they were able to show that pathogenic Tau is able to broadly impair lysosomal function ahead of Tau accumulation. They also show that the degradative function of lysosomes, but not their motility, can be restored through pharmacological activation of autophagy, leading to reduced Tau levels. This work opens new therapeutic opportunities to eliminate early‐on pathological misfolded Tau proteins before they can engage in a vicious cycle of aggregation, amplification and propagation.

## Graphical abstract

Tauopathies are neurodegenerative diseases characterized by misfolded tau accumulation and lysosomal dysfunction. Pathogenic mutant Tau stalls lysosomes in iPSC‐derived neurons upstream of Tau accumulation. Pharmacological activation of autophagy activation reduces Tau burden without rescuing lysosome motility, revealing that trafficking and degradation are separable axes of lysosomal biology.

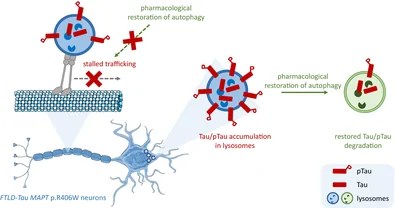

The microtubule‐associated protein tau (MAPT) plays important roles in neurons by promoting the assembly and stability of microtubules in axons. Six Tau isoforms resulting from alternative mRNA splicing are expressed in the adult brain [1]. Their functions are modulated by many post‐translational modifications including phosphorylation at multiple residues [2, 3]. In a number of neurodegenerative diseases—named tauopathies and that include Alzheimer's disease (AD), Pick's disease (PiD), chronic traumatic encephalopathy (CTE), argyrophilic grain disease (AGD), corticobasal degeneration (CBD) and progressive supranuclear palsy (PSP) to name but a few—Tau accumulates as hyperphosphorylated misfolded species that ultimately aggregate and form filamentous tangle inclusions within the soma, dendrites and axons [1, 4]. Tau aggregates were proposed to amplify and propagate in a prion‐like manner that correlates with the staging of Tau pathology and the evolution of clinical symptoms [1, 4]. Most tauopathies are sporadic, but familial mutations in Tau alter the structural and aggregation properties of Tau proteins and cause autosomal dominant forms of frontotemporal lobar dementia with Tau inclusions (FTLD‐Tau) [1, 4].

As non‐proliferating long‐lived cells, neurons are particularly vulnerable to defects in protein quality control and clearance mechanisms that help maintaining proteostasis, such as the autophagy–lysosome pathway. In neurons, axonal autophagosomes are transported along microtubules to the soma where they mature into degradative acidic autolysosomes, resulting in cargo degradation. Lysosomal and autophagic dysfunctions are frequently observed in tauopathies, yet whether they are a cause or a consequence of misfolded and aggregated Tau accumulation is not clear.

In a recent study, Mirfakhar et al. used human iPSC‐derived neurons harboring *MAPT* p.R406W, a disease‐associated missense mutation in the *MAPT* gene located outside the microtubule‐binding domain, to address this issue and investigate how endogenous pathogenic Tau impacts the autophagy–lysosome system in a physiopathologically relevant context [5].

The authors made a number of interesting observations, showing that a distinct localization of different Tau species within the lysosomes could affect their specific clearance, and that lysosomal degradative function and trafficking could be dissociated for further therapeutic strategies aiming at decreasing toxic protein accumulation (Figure 1). Using super‐resolution imaging, the authors showed a marked difference in the localization of total Tau and phosphorylated Tau (pTau) in the lysosomes of wild‐type and *MAPT* p.R406W neurons, with the latter exhibiting an even more pronounced effect. Whereas total Tau was predominantly localized to the lumen of lysosomes, in agreement with its ongoing degradation, pTau was mostly stalled at the membrane of lysosomes and resisted clearance (Figure 1). This indicates that some post‐translational modifications of Tau may act as barriers against the degradative action of lysosomes. Accumulation of pTau at the membrane of lysosomes may “clog” them and impair their proper functioning, for instance by reducing protein delivery to lysosomes via autophagosome‐lysosome fusion, in agreement with the increased lysosomal volume, the defect of trafficking and fusion with autophagosomes as well as the accumulation of undigested material observed in mutant neurons (Figure 1) [5]. Furthermore, the expression of many genes involved in phagosome maturation, regulation of autophagy and autophagosome and vesicle transport was shown to be significantly altered in *MAPT* p.R406W neurons. However, the way in which this transcriptional response is coupled to the reported effects of tau on lysosomes remains unclear. While the underlying mechanisms need further investigation, neurons expressing *MAPT* p.R406W accumulated total Tau and pTau in lysosomal compartments, triggering a vicious cascade leading to impaired lysosomal degradation and defective autophagy–lysosome pathway. Lysosomal dysfunction appears to be an early consequence of pathological mutations in Tau, preceding its aggregation. Importantly, although only partially, pharmacological enhancement of autophagic flux restored lysosomal degradation and reduced Tau burden but did not rescue the defects in lysosomal motility (Figure 1). Lysosomal trafficking and degradative functions therefore appear to constitute two independent components in neuronal lysosomal dynamics that could be selectively modulated [5].

**FIGURE 1:**
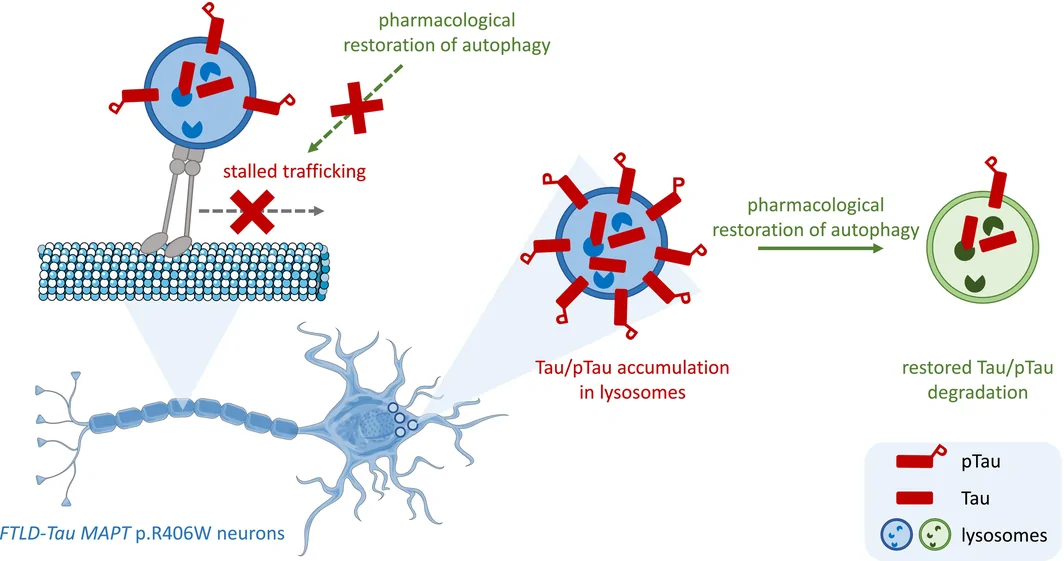
Schematic summary of autophagy‐lysosomal defects in human iPSC‐derived neurons expressing mutated *MAPT* p.R406W Tau (adapted from [5]). Details are in the main text (“P” symbols represent Tau protein phosphorylations). This figure was created using image(s) provided by Servier Medical Art (https://smart.servier.com), licensed under CC BY 4.0 (https://creativecommons.org/licenses/by/4.0/).

The work highlighted here opens new therapeutic opportunities to pharmacologically target and restore proteostasis, in order to eliminate early‐on pathological misfolded Tau proteins before they can engage in a vicious cycle of aggregation, amplification and propagation. Given the extent of Tau post‐translational modifications, both under physiological and pathological conditions, as well as the numerous reported mutations that can cause FTLD‐Tau, future investigations are needed to better understand how Tau interacts with cellular clearance mechanisms, in particular the mechanisms at play for its retention at the lysosomal membrane and how this is toxic for proper lysosomal function. Misfolding, aggregation and propagation of specific proteins is not restricted to tauopathies, but rather a common pathological hallmark in many neurodegenerative diseases. The pathological proteins involved, such as α‐synuclein in Parkinson's disease (PD), multiple system atrophy (MSA) and Dementia with Lewy Bodies (DLB), TDP‐43 in amyotrophic lateral sclerosis (ALS) and FTLD‐TDP, or amyloid precursor protein (APP) in AD, were also associated with dysfunctions in the autophagy–lysosome system. Similarly to what is known for Tau, the functions and properties of these proteins are also modulated by post‐translational modifications and by familial mutations. Of particular interest would be investigating whether the pathological forms of these proteins can also mislocalize within lysosomes, and impair their distribution and degradative capacities. It would also be interesting to determine whether these defects could be rescued through the pharmacological enhancement of autophagy. This line of investigation may lead the way to tremendous therapeutic opportunities targeting very early events in a wide range of neurodegenerative diseases for which no definitive treatment is available.

## Funding

The authors have nothing to report.

## Conflicts of Interest

The authors declare no conflicts of interest.

## Data Availability

Data sharing not applicable to this article as no datasets were generated or analysed during the current study.
